# Sample size calculation

**DOI:** 10.4103/0974-7788.64395

**Published:** 2010

**Authors:** Younis Abed AL-Wahhab M. Skaik

**Affiliations:** *Department of Laboratory Medicine, Faculty of Applied Medical Sciences, AL Azhar University-Gaza, Palestine* E-mail: Y_skaik@hotmail.com

Sir,

Regarding the research methodology paper entitled "Sample size calculation" by Kadam and Bhalerao,[1] which was published recently in IJAR, I would like to point out that the formula described in the article can be used in simple random sampling. Hence, authors should have mentioned this in their article.

I would also like to point out that in some cases one can screen and study the whole target population contrary to what is said at the beginning of the fi rst paragraph. "It is naturally neither practical and feasible to study the whole population in any study." For example, the study which assessed the possible association of bronchial asthma with the inhalation of volatile acids in dark rooms amongst the radiographers evaluated the whole population then being a total of 100 radiographers in the Gaza Strip in Palestine[2] and all were studied.
